# A Case of Acute Posterior Multifocal Placoid Pigment Epitheliopathy Associated With Anterior Ischemic Optic Neuropathy

**DOI:** 10.1155/crop/6676190

**Published:** 2026-10-22

**Authors:** Marie Ikeda, Toshiyuki Oshitari, Tomohiko Usui

**Affiliations:** ^1^ Department of Ophthalmology International University of Health and Welfare Narita Hospital Narita City Chiba Japan; ^2^ Department of Ophthalmology and Visual Science Chiba University Graduate School of Medicine Chiba Japan chiba-u.ac.jp

**Keywords:** acute posterior multifocal placoid pigment epitheliopathy, nonarteritic anterior ischemic optic neuropathy, oral prednisone, the short posterior ciliary artery

## Abstract

**Introduction:**

We report a case of unilateral presumed nonarteritic anterior ischemic optic neuropathy (NAION) associated with acute posterior multifocal placoid pigment epitheliopathy (APMPPE).

**Case Presentation:**

A 59‐year‐old female presented with blurred left vision for 9 days. At the initial examination, her best‐corrected visual acuities (BCVAs) were 20/16 OD and 20/30 OS. No signs of inflammation were observed in the anterior chamber or vitreous. The critical fusion frequency (CFF) was 38 Hz in OD and 27 Hz in OS. Fundus examination revealed optic disc edema in the left eye and multiple placoid lesions in both eyes. Optical coherence tomography showed a serous retinal detachment (SRD) beneath the macula of the left eye. Humphrey field analysis revealed visual field defects in the inferonasal sectoral area at the left eye and mild concentric constrictions in both eyes. Fluorescein angiography revealed early hypofluorescence and late hyperfluorescence in the superotemporal region of the left optic nerve swelling and placoid lesions in both eyes. Indocyanine green angiography revealed multiple hypofluorescent spots in both eyes from the early to late phases. Based on the above findings, a diagnosis of APMPPE complicated by left presumed NAION was made, and oral prednisolone 30 mg/day was started. After 2 months, the left BCVA improved to 20/16, CFF recovered to 37 Hz and SRD and optic nerve edema disappeared. Mild concentric constrictions improved in both eyes but the inferonasal sectoral visual field defects persisted in the left eye; the left nerve fibre layer thinning appeared to be consistent with the remaining inferonasal sectoral visual field defects.

**Discussion:**

We encountered a case of APMPPE complicated by presumed NAION. Inflammatory vascular occlusion of the short posterior ciliary artery may be involved in the development of NAION.

## 1. Introduction

Acute posterior multifocal placoid pigment epitheliopathy (APMPPE) is a rare inflammatory disease characterized by multiple white dots in the retina. Relatively young people are affected, and the proportions of men and women are almost equal. The symptoms are usually bilateral and have an acute onset. Placoid lesions are characterized by hypofluorescence in the early phase and hyperfluorescence in the late phase on fluorescein angiography (FA).

In contrast, ischemic optic neuropathy is caused by inadequate blood supply to the optic nerve, which results in impaired vision. Anterior ischemic optic neuropathy (AION) is caused by ischemia of the optic nerve head supplied by the short posterior ciliary arteries [1]. AION is classified into two types: arteritic anterior ischemic optic neuropathy (AAION) caused by vasculitis, such as giant cell arteritis, and nonarteritic anterior ischemic optic neuropathy (NAION) caused by conditions other than vasculitis. Major risk factors for NAION include diabetes, hypertension and dyslipidemia [2]. However, in a search of PubMed, no reports of APMPPE accompanied by NAION have been published in English literature.

We report a rare case of APMPPE associated with presumed NAION in the left eye, which improved during follow‐up after oral prednisolone (PSL) treatment.

## 2. Case Presentation

### 2.1. Subjective

A 59‐year‐old female presented with blurred vision for 9 days and was diagnosed with left optic neuritis at another hospital. Intravenous methylprednisolone pulse therapy was recommended, but the patient refused. The patient was then referred to our hospital for further examination and treatment. Her medical history included gastric submucosal tumour and gastroesophageal reflux disease. She had no history of diabetes, hypertension or dyslipidemia. She had common‐cold symptoms 1 week before presentation. There was no poliosis. She had occasional headaches, but no tinnitus. She had a Covid‐19 infection and was vaccinated 1 year ago. She had no pets at the time. She had no jaw claudication, temporal artery tenderness, polymyalgia rheumatica symptoms including fever, weight loss and systemic malaise. She had no pain with eye movement and no dyschromatopsia. There were no findings of small disc or crowded disc in the fellow eye.

### 2.2. Objective

At the first examination, her best‐corrected visual acuities (BCVAs) were 20/16 OD and 20/30 OS, with no signs of inflammation in the anterior chamber or vitreous. The intraocular pressure was 16 mmHg OD and 15 mmHg OS. The left pupil was slightly dilated. The light reflex was prompt and complete in the right eye, but dull and incomplete in the left eye. A relative afferent pupillary defect (RAPD) was absent in the right eye, but present in the left eye. The critical fusion frequencies (CFF) were 38 and 27 Hz OD and OS, respectively. Fundus examination revealed optic disc edema in the left eye and multiple white placoid lesions in both eyes (Figure 1). Fundus autofluorescence (FAF) images indicated hyperautofluorescent lesions corresponding to the multiple white placoid lesions (Figure 2). Swept‐source optical coherence tomography (OCT) revealed a serous retinal detachment (SRD) under the macula of the left eye (Figure 3). Humphrey field analysis (HFA) revealed visual field defects in the inferonasal sectoral area at the left eye and mild concentric constrictions in both eyes. However, there were no central scotomas in both eyes (Figure 4). FA showed hypofluorescence in the superotemporal region of the left optic nerve and placoid lesions in the early phase and hyperfluorescence in the same lesions in the late phase (Figure 5). Indocyanine green angiography (ICGA) revealed multiple hypofluorescent spots in both eyes from the early to late phases (Figure 6). Magnetic resonance imaging (MRI) showed no obvious inflammation of the optic nerves on either side (Figure 7). There were no findings of acute cerebral infarction, hemorrhage or mass lesions. Serum blood tests revealed no inflammatory findings, including C‐reactive protein, erythrocyte sedimentation rate (ESR), white blood cell number or infectious findings, including viral hepatitis, human immunodeficiency virus and syphilis. Tests for antiaquaporin 4 antibody and antineutrophil cytoplasmic antibody were negative. Human leukocyte antigen‐DR4 (HLA‐DR4) was negative.

**Figure 1:**
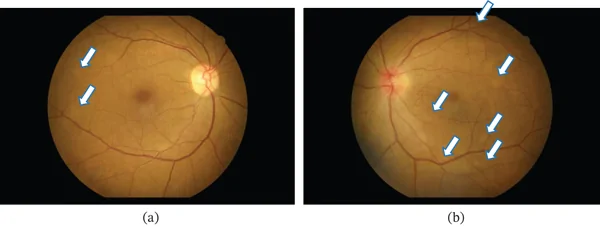
Fundus imaging of the (a) right eye and the (b) left eye. Colour fundus photographs indicated multiple placoid lesions (indicated by arrows) in the posterior pole and left optic disc swelling.

**Figure 2:**
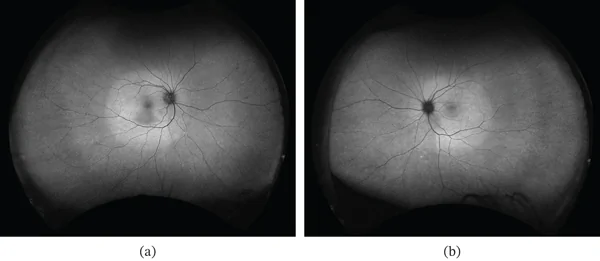
Fundus autofluorescence (FAF) of the (a) right eye and the (b) left eye. FAF indicated hyperautofluorescent lesions corresponding to the multiple placoid lesions.

**Figure 3:**
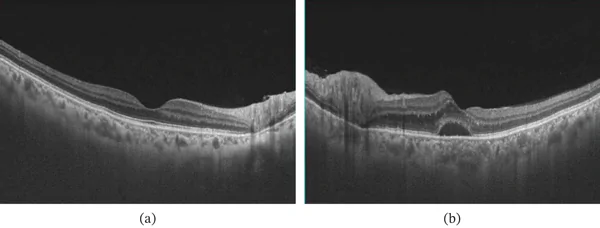
OCT of the (a) right eye and the (b) left eye. Optic disc and macula edema with serous retinal detachment were found in the left eye. Thickening of choroidal membranes was found in the left but not observed in the right.

**Figure 4:**
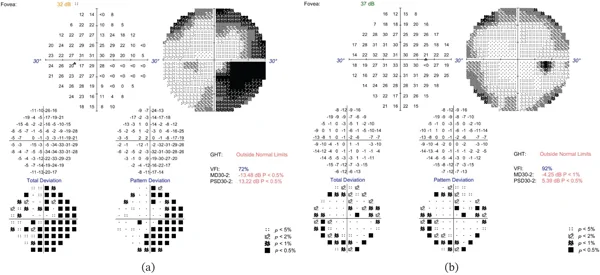
Humphrey field analysis (HFA) of the (a) left eye and the (b) right eye. HFA revealed visual field defects in the inferonasal sectoral area of the left eye and mild concentric constrictions in both eyes. However, there were no central scotomas in either eye.

**Figure 5:**
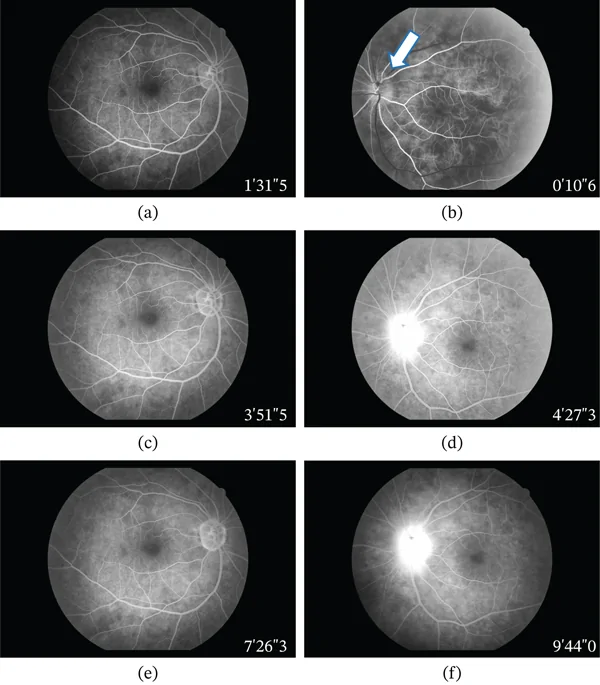
Fluorescein angiography (FA) from the early phase to the late phase of the (a,c,e) right eye and the (b,d,f) left eye. FA revealed superotemporal region of the left optic nerve and placoid lesions with hypofluorescence in the early phases and hyperfluorescence in the late phases. Sectoral optic nerve hypoperfusion in early FA showed the ischemic sign of the left optic nerve. Early hypofluorescence and late hyperfluorescence corresponding to the placoid lesions indicated the evidence of choriocapillaris involvement.

**Figure 6:**
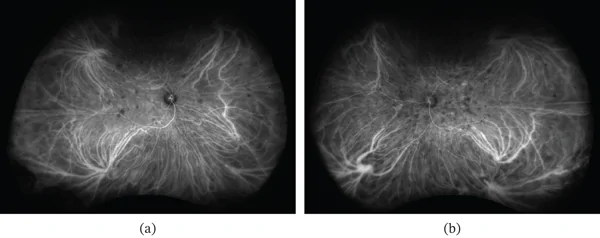
Indocyanine green angiography (ICGA) of the (a) right eye and the (b) left eye. Multiple hypofluorescence spots were found in both eyes from early to late phases. These findings of ICGA indicated the evidence of choriocapillaris involvement.

**Figure 7:**
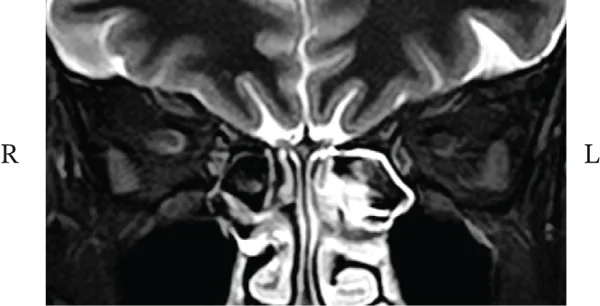
MRI findings of T2 short T1 inversion recovery (STIR). The patient did not agree with using contrast agents and thus we have no gadolinium‐enhanced MRI findings. However, no abnormal findings of the optic nerves were seen on STIR MRI images. However, the absence of abnormalities on STIR imaging should not be interpreted as definitively excluding inflammatory optic neuritis.

### 2.3. Assessment

Based on these findings, including the FA/ICGA, the final diagnosis was APMPPE associated with left presumed NAION.

### 2.4. Plan

Oral PSL (30 mg/day) was initiated and tapered over 3 months. The placoid lesions gradually disappeared within 2–3 weeks in both eyes; however, hard exudates remained in the left eye (Figure 8a,b). The hyperautofluorescent lesions in FAF also disappeared (Figure 8c,d). The left SRD and optic disc edema improved during follow‐up after oral PSL treatment (Figure 8e). After 2 months, the left BCVA increased to 20/16, and the left CFF recovered to 37 Hz. However, a left nerve fibre layer defect (NFLD) appeared in the temporal area, and inferonasal sectoral visual field defects corresponding to the hypofluorescent area of the left optic nerve in the early phases of FA persisted after the treatment (Figure 8f–h). The remaining visual field defects were anatomically related to the thinning of retinal nerve fibre layer (RNFL) and ganglion cell complex (GCC) thicknesses after the treatment (Figure 8f–h). In addition, at the final visit, the inferonasal sectoral visual field defects in HFA persisted in the left eye even after the steroid treatment, and the findings of visual field tests were corresponding to the thinning of RNFL and GCC thicknesses (Figure 9). This time course changes of the optic nerve atrophy supported that the sectoral hypofluorescence area in FA showed the ischemic lesions and that the patient had NAION associated with APMPPE (Figure 9).

**Figure 8:**
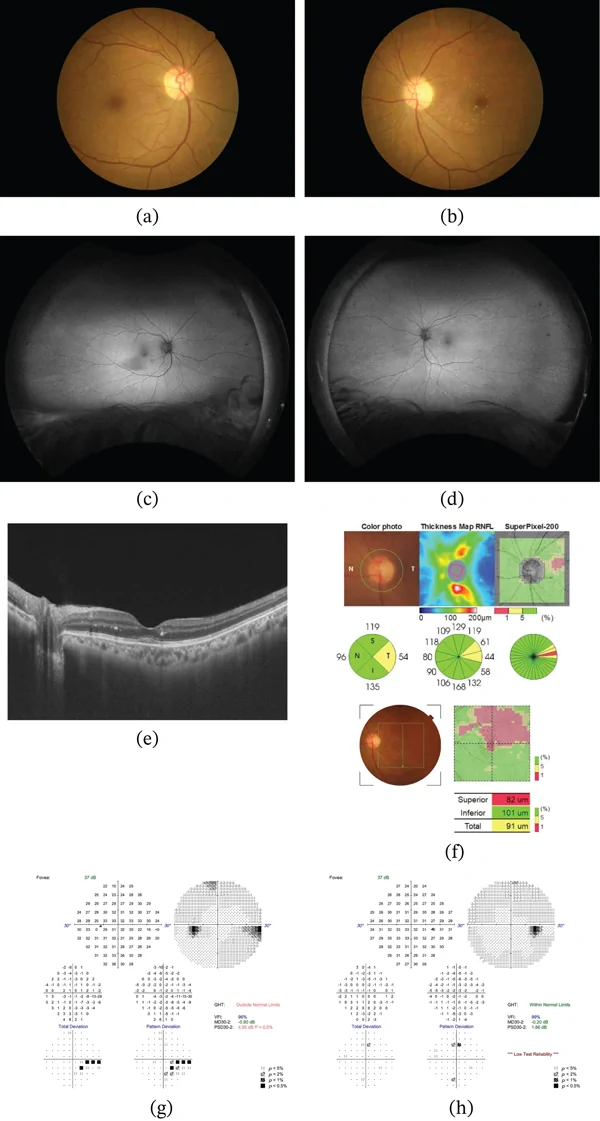
Fundus image of the (a) right eye and the (b) left eye after treatment. Placoid lesions and left optic disc edema resolved. No small and/or crowded discs were found. Fundus autofluorescence (FAF) of the (c) right eye and the (d) left eye after treatment. Hyperautofluorescent lesions in FAF disappeared. Macula OCT of the (e) left eye. Serous retinal detachment improved. The retinal nerve fibre layer (RNFL) thickness and ganglion cell complex (GCC) of the (f) left eye. The left nerve fibre layer thinning appeared to correspond to the sectoral superotemporal hypofluorescent area of the left optic nerve during the early phases of FA. HFA of the (g) left eye and (h) right eye after 3 months. Mild concentric constrictions improved in both eyes during follow‐up after oral PSL treatment but the inferonasal sectoral field defects persisted in the left eye even after the steroid treatment. The remaining visual field defects at the left eye were corresponding to the hypofluorescence area of the left optic nerve in the early phase of FA. In addition, the remaining visual field defects were anatomically related to the thinning of RNFL and GCC thicknesses after the treatment.

**Figure 9:**
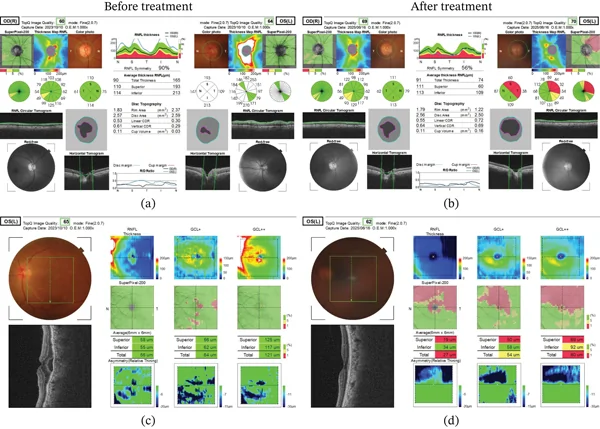
(a,b) Retinal nerve fibre layer (RNFL) thicknesses and (c,d) ganglion cell complexes (GCC) (a,c) before and (b,d) 2 years after steroid treatment. Before treatment, the left optic disc swelling was observed but the swelling was not so strong and thus, early hypoperfusion of FA was unlikely to be caused by a masking effect of optic disc edema or inflammatory material. After 2 years of treatment, the (b) significant RNFL thinning and the (d) GCC thinning corresponding to the superotemporal sectoral hypoperfusion area was observed (red colour area). These clinical courses of OCT findings supported that the early sectoral hypoflorescence in FA was related to ischemic damage in the left optic nerve.

Informed consent was obtained from the patient for publication of her clinical details and images.

## 3. Discussion

APMPPE was first described by Gass in 1968 as a self‐limiting inflammatory disease involving the retina and the choroid [3]. Initially, the retinal pigment epithelium was thought to be the main site of inflammation; however, later, the choriocapillaris was thought to be the site of inflammation [4]. Its pathogenesis is still unknown, but autoimmunity induced by a preceding viral infection may be, in part, associated with the pathogenesis of APMPPE. In the present case, the preceding infection was suspected to have triggered the inflammatory response.

A possible differential diagnosis of the retinal condition was Vogt‐Koyanagi‐Harada disease (VKH); however, VKH was thought to be unlikely because of the absence of choroidal thickening in the right eye, despite the acute stage of the disease. In addition, HLA‐DR4 was negative, and the patient did not have tinnitus and poliosis. In Japan, there was a case report of VKH accompanied with NAION but in this case, optic disc swelling persisted for over 5 months and the case had various risk factors including small and crowded discs, hypertension and hyperlipidemia [5]. Our case had no apparent risk factors for NAION and optic disc edema was relatively mild. The placoid lesions in our case demonstrated early hypofluorescence followed by late hyperfluorescence. These findings were in contrast with multiple evanescent white dot syndrome (MEWDS), the placoid lesions of MEWDS typically exhibit hyperfluorescence in the early phase and late staining. In serpiginous choroiditis, characteristic snake‐like or geographic chorioretinal fused lesions are not found; thus, it did not match this case. Neuroretinitis caused by cat‐scratch disease was considered unlikely due to the absence of contact history. Optic neuritis secondary to other diseases was also deemed unlikely based on MRI findings and blood test results. Furthermore, no inflammatory findings, including ESR, were observed in the blood tests and no other symptoms related to AAION were found; thus, AION was nonarteritic rather than arteritic.

Several studies have been published on papillitis in APMPPE [4, 6–9]. The difference between papillitis and this case was that the left optic nerve had a sectoral hypofluorescent lesion in the early phase of FA, suggesting decreased blood supply. Although retinal and choroidal lesions of APMPPE improved during the follow‐up after oral PSL treatment, NFLD and corresponding visual field defects remained because the ischemic optic nerve damage caused by NAION was irreversible.

Although APMPPE may resolve spontaneously, this case involved NAION and vascular inflammation was thought to contribute to the development of NAION. Therefore, we selected oral steroid treatment for NAION. Because there were no central scotomas, we did not select pulse therapy but oral PSL based on an empirical decision.

There were two previous cases of APMPPE with NAION in Japanese literature [10, 11]. One patient chose corticosteroid pulse therapy as the treatment method, and the other received oral steroids 2 months after oral aspirin treatment. In both cases, as in this case, the visual field defects slightly improved but persisted. Both the optic nerve head and the posterior choriocapillaris are supplied by the short posterior ciliary arteries. Since visual field improvement was achieved with oral steroids, inflammatory vascular occlusion of the short posterior ciliary artery may have played a partial role in the pathogenesis of NAION. Similar to two Japanese cases of APMPPE with NAION, our case may support the concept of inflammatory vascular compromise extending beyond the choriocapillaris since both the choriocapillaris and optic nerve head are supplied by the short posterior ciliary circulation. However, we must acknowledge that the distinction between inflammatory optic disc neuropathy and secondary ischemic injury may not always be absolute in this case.

There is another possible pathogenesis of APMPPE accompanied by NAION. A recent multimodal imaging reveals that the pathogenesis of APMPPE may be related to profound choriocapillaris ischemia induced by primary inflammatory inner choroid vascular occlusive process [12]. In fact, some patients with APMPPE accompanied by an occlusive vasculitis affecting the central nervous system because vessels share some antigens with those of the choriocapillaris [13]. Thus, patients with APMPPE may have an inflammatory process occurring at the level of the choriocapillaris, similar to targeting elements of the vessels, resulting in occlusion of the short posterior ciliary arteries.

Herein, we report a rare case of APMPPE accompanied by presumed NAION. Although APMPPE has a good visual prognosis, NAION is resistant to treatment. Thus, clinicians should note that NAION, which has a poor visual prognosis, can be accompanied by APMPPE as both the choriocapillaris and optic nerve head are primarily supplied by the short posterior ciliary arteries.

The corresponding author affirms that this manuscript is an honest, accurate and transparent account of the study being reported; that no important aspects of the study have been omitted; and that any discrepancies from the study as planned have been explained.

## Funding

This study was supported by a grant‐in‐aid from the Ministry of Education, Science, Sports, and Culture of the Japanese Government (22K09829) and a grant‐in‐aid from the Eye Research Foundation for the Aged in Japan.

## Disclosure

All authors have read and approved the final version of the manuscript. Corresponding author, Toshiyuki Oshitari, had full access to all of the data in this study and takes complete responsibility for the integrity of the data and the accuracy of the data analysis.

## Consent

The patient provided consent for publication of her clinical details and images.

## Conflicts of Interest

The authors declare no conflicts of interest.

## Data Availability

The data supporting the findings of this study are available from the corresponding author upon reasonable request.
